# The Relative Value of the Cuspidati

**Published:** 1882-06

**Authors:** F. A. Brewer


### THE RELATIVE VALUE OF THE CUSPIDATI.

BY F. A. BREWER, D.D.S.

I frequently am disposed to wonder why the importance of these special organs of defense is not cherished with that true regard they so richly deserve, by more of the members of our profession. Their sacrifice is so frequent, not alone by the more indiscreet, but also by the more deserving. Such recognized carelessness cannot prove otherwise than painful to the more sensitive pathologist. Close and persistent observation, as well as analogical comparison of these special teeth with the remaining series, must convince one's better judgment of an additional obligatory mission on their part, in their work of the life, as well as that of the functional subserviency of facial prominence, and organs of comminution. If one will but exercise his perceptive faculties properly, he will agree with the author, that the above function is not only fulfilled, but in addition thereto, they serve as special wedging "pins," not only for the normal retention of the anterior teeth, but also for those more posterior, as well as exhibit their prowess as special protectors in resisting both concussive and disintegrating influences. In order that we may substantiate the grounds for such claims, as well as to offset opinions derogatory thereto, we will endeavor to present some important reasons as a basis for our argument: 1. Common sense teaches one that the position these organs occupy within the arch of the maxilla is one where there must naturally exist a greater combating occludent force than elsewhere throughout the arch. 2. By their increased length of fang-surface, through a greater depth of implantation within the jaw, thereby giving them firmer support, they are better enabled to withstand the ordinary concussions, than their more anterior neighbors of the incisive class. Again: the additional length of these teeth, as found in the superior jaw, by which nature anticipated a greater strain brought to bear upon the region of the superior arch, as compared with the inferior, confirms our conclusions with a degree of certainty. 3. It will be noticed, moreover, that the prominent convexity of their lingual and labial surfaces teaches plainly the wise forethought which nature's God had, in providing the necessary bulk of enamel and "dentos," to be acted upon previous to an impact occurring to their more ill-conditioned incisive neighbors. Why, sirs, their very typical character throughout points most conclusively to such evidence. Again is this shown by the delay in their eruptive period, in many cases by not making an appearance until a majority of the whole series have fully erupted; and in some instances, not until their predecessors, the second molars, had not only appeared, but had even assumed their true position in the arch. A safe hypothesis is thus formed, that nature so designed, in order to serve as prime factors in the duration of the original whole series, not only through ordinary life, but to a *ripe old age*. But, notwithstanding the above reasons should suffice to convince an ordinary mind of the justness of our plea for the preservation of these teeth, we would say, that we have still further evidence in substantiation of our theory, as furnished by the occasional occurrence of cases in our own practice, relating to the evil effects of the early loss of the incisors of the superior jaw, induced through a premature removal of the cuspidati. As two cases are fresh within our memory, we will refer to them. In one case, aged 44, a diagnosis revealed the disintegration of the entire osseous integuments, embracing the whole length of the labial alveolar region, occupied by the roots of both superior central incisors, as well as the mesial septa, and a part of their lingual investments. Also did we find an absence of the mesial septa and a part or quite one-half of the outer walls of the adjoining laterals, as well as a portion of their lingual attachments. All were loose, elongated, and thrown far beyond their normal limits; and in fact, were merely sustained by their ligamentous tissue.

Another case, aged 47, came under our care, with the loss of nearly or quite two thirds of the labial and mesial walls of both superior central teeth; also a part of the mesial septa of both adjoining laterals. All four were loose, and suffering from periosteal congestion. The two centrals were not only elongated, but also, like case No. 1, thrown forward entirely from their normal axes. In both of these eases we found the canines absent, having been extracted soon after making their appearance. As they did not erupt until the fourteenth year from birth, of course I found their usually assigned position in the arch closed and occupied by the first bicuspids. The cuspidati, in both cases, had been sacrificed upon the grounds that the bicuspids were of more intrinsic value as masticators. On examination, the lingual surfaces of the incisors affected were found to be much worn by attrition, as was also the cutting edge of the inferior incisors, and coronary portions of the canine and bicuspid family of both jaws. In one case, the patient dates the commencement of this trouble—that of soreness of both the centrals at their basis—at about the age of 32; in the other case, at the age of 35 or 37. The services of the family dentist in both cases came early into play; and the scaler and scalpel frequently indulged in, afforded but temporary relief. Finally, finding the basilar disintegration continued, both cases had been abandoned, upon the ground that the primary lesion originated from a constitutional cause. Our diagnosis failed to corroborate this opinion. These patients were both stout, healthy, and of a vigorous type of manhood. Had there existed other indications, or a presentment of mineral poison, such manifestations could have been better observed in the posterior organs, by a looseness of the *dens sapientiœ,* and second molars; but these teeth were found firm and normal. So with the adjoining series. All questions relative to undue luxation of the teeth affected were answered in the negative. In fact, the diagnosis of both cases was sufficient to convince the most skeptical of the fact that the true primary cause came from the absence of the cuspidati. Still there may be a difference of opinion relative to such effects being based on constitutional grounds. However, the fact will not be denied, that in those cases of scissors-form articulation arising from abrasive influences, even where the full complement of the teeth is normal, there will be observed a loss not merely of the cusp, but fully one-third or more of the coronary portion of the cuspidati, previous to an attack upon the incisors. Also may be noticed the same phenomena in cases of non-interlocking, and when the whole dental series become involved. Again: it cannot be denied, that by the presence of these special auxiliaries, an undue anterior articulating motion is more or less arrested, and the concussive force, so fatal to the incisors, greatly lessened, thus preserving the labial maxilliary integuments to an indefinite period. More noticeable will the intrinsic value of their assistance be observed in cases of absence of the central teeth, second bicuspids, or first and second molars of each jaw. It must be borne in mind, however, that in our remarks we make no reference to those cases of interlocking where the diametral proportions of crown surface, both in breadth and depth, are equal; but more especially to those organs whose transverse diameters reach three-eighths or nearly one-half inch, and whose crown length equals or even exceeds the length of its root, which type of tooth, we are well aware, is unable to withstand the great strain brought to bear upon it, as in the former class. Few are known to do duty free from abrasion for the space of twenty five or even twenty years. Therefore, they need all the support possible to maintain their basilar integuments. Hence, much nicety of judgment becomes necessary when called upon to sacrifice the cuspidati. Let me ask, that before the forceps are applied for carrying out this fatal design, that we stop and ask ourselves the question, What damage shall we inflict to the remaining series by such sacrifice? If decayed seriously, can we not restore to such shape that it will serve better in combating articulating forces than an artificial substitute, which we know so readily yields upon pressure through the congested state of the rugæ? Surely, a pivoted tooth is much superior. It is a lamentable fact, that the progenitors of our profession have failed in their teachings to impress upon the minds of the less thoughtful, as well as the new beginner, the importance of looking well to the defense and preservation of the cuspidati, in order to prolong the existence of the series to an advanced age—a period certainly when the presence of the whole series is as desirable as during youth or middle age. Who will question, that an old man does not need his teeth as well as a young man? Does he not feel just as proud of them, if not more so, especially if they are not diseased? Should we not try, therefore, to preserve them for him in his old days? Will he not look to us as the natural guardians for their preservation? If not, to whom? And in order that our professional ability may not be questioned by the world at large, we should, as practitioners of a beneficent art, give more attention to the needs of an important condition as the one under consideration. So partial are we to the value and necessary presence of the cuspidati, that even though their eruption should not occur until the twentieth year from birth, we should, under all circumstances, sacrifice one of the bicuspid family, either sound or unsound, whether the arch be crowded or not.

The oral series has been likened unto a Pandora's box. Verily, are not the cuspidati the keys that lock it?—*Trans. Cal. State Dental Association.*

---

"How are you and your wife coming on?" asked a Galveston man of a colored man. "She's run me off, boss." "What's the matter?" "I is to blame, boss. I gave her a splendid white silk dress, and den she got so proud, she had no use for me. She 'lowed I was too dark to match de dress."

